# Evolution of white matter damage in amyotrophic lateral sclerosis

**DOI:** 10.1002/acn3.51035

**Published:** 2020-05-04

**Authors:** Matt C. Gabel, Rebecca J. Broad, Alexandra L. Young, Sharon Abrahams, Mark E. Bastin, Ricarda A. L. Menke, Ammar Al‐Chalabi, Laura H. Goldstein, Stella Tsermentseli, Daniel C. Alexander, Martin R. Turner, P. Nigel Leigh, Mara Cercignani

**Affiliations:** ^1^ Department of Neuroscience Clinical Imaging Sciences Centre Brighton and Sussex Medical School University of Sussex Brighton East Sussex United Kingdom; ^2^ Department of Neuroscience Trafford Centre Brighton and Sussex Medical School University of Sussex Brighton East Sussex United Kingdom; ^3^ Centre for Medical Image Computing Department of Computer Science University College London Gower Street London WC1E 6BT United Kingdom; ^4^ Department of Psychology School of Philosophy, Psychology & Language Sciences Euan MacDonald Centre for Motor Neurone Disease Research University of Edinburgh Edinburgh United Kingdom; ^5^ Wellcome Centre for Integrative Neuroimaging FMRIB Nuffield Department of Clinical Neurosciences University of Oxford Oxford United Kingdom; ^6^ Nuffield Department of Clinical Neurosciences University of Oxford Oxford United Kingdom; ^7^ Department of Basic and Clinical Neuroscience King's College London Maurice Wohl Clinical Neuroscience Institute London United Kingdom; ^8^ Department of Neurology King’s College Hospital London United Kingdom; ^9^ Department of Psychology Institute of Psychiatry, Psychology, and Neuroscience King's College London London United Kingdom; ^10^ Faculty of Education and Health University of Greenwich London United Kingdom

## Abstract

**Objective:**

To characterize disease evolution in amyotrophic lateral sclerosis using an event‐based model designed to extract temporal information from cross‐sectional data. Conventional methods for understanding mechanisms of rapidly progressive neurodegenerative disorders are limited by the subjectivity inherent in the selection of a limited range of measurements, and the need to acquire longitudinal data.

**Methods:**

The event‐based model characterizes a disease as a series of events, each comprising a significant change in subject state. The model was applied to data from 154 patients and 128 healthy controls selected from five independent diffusion MRI datasets acquired in four different imaging laboratories between 1999 and 2016. The biomarkers modeled were mean fractional anisotropy values of white matter tracts implicated in amyotrophic lateral sclerosis. The cerebral portion of the corticospinal tract was divided into three segments.

**Results:**

Application of the model to the pooled datasets revealed that the corticospinal tracts were involved before other white matter tracts. Distal corticospinal tract segments were involved earlier than more proximal (i.e., cephalad) segments. In addition, the model revealed early ordering of fractional anisotropy change in the corpus callosum and subsequently in long association fibers.

**Interpretation:**

These findings represent data‐driven evidence for early involvement of the corticospinal tracts and body of the corpus callosum in keeping with conventional approaches to image analysis, while providing new evidence to inform directional degeneration of the corticospinal tracts. This data‐driven model provides new insight into the dynamics of neuronal damage in amyotrophic lateral sclerosis.

## Introduction

Amyotrophic Lateral Sclerosis (ALS) is now recognized as both a clinically and pathogenically heterogeneous disorder.^1^ This poses new challenges for understanding disease evolution in relation to the underlying molecular and cellular mechanisms of neurodegeneration.

Thus far, schemes for studying the evolution and staging of these diseases have depended on the selection of phenotypic and biomarker criteria (broadly defined to include a wide range of clinical, molecular, and neuroimaging measures). Examples include the King’s and MiToS systems for clinical in vivo staging^2^, ^3^ that predefine milestones representing stages by involvement of body regions and by functions. The Braak postmortem histopathological staging schemes^4^, ^5^ applied to Alzheimer’s disease and ALS, depend on a priori assumptions on the pattern of pathological change, which are by definition end‐stage. In this latter context, the notion of stage is necessarily an inference from the observed patterns of pathological change. The same caveat applies to neuroimaging staging systems that (for example) select certain tracts for analysis (e.g.^6^) based on similar a priori assumptions derived from the postmortem model. Subjective inferences must be drawn regarding disease spread, since it is conceivable that the areas in which changes appear over time were affected at baseline, but at a level below detection by the techniques applied. In other words, there is a problem of sensitivity and selection of criteria of change, even in longitudinal neuroimaging studies. In addition, missing data^7^, ^8^ is likely to introduce bias into longitudinal analyses since missingness is unlikely to be random in ALS.^9^

In order to address these problems, a number of data‐led approaches have been developed, but are as yet not integrated into prospective phenotyping studies or clinical trials. In ALS, a data driven study using latent class cluster analysis has identified prognostic and clinical subgroups that are distinct from those derived from analyses based on subjective clinical subgroupings.^10^ More ambitious data‐driven models have been developed to understand the sequence of biomarker changes in Alzheimer’s disease^11^, ^12^, ^13^, ^14^, ^15^ and Huntington’s disease:^11^ probabilistic generative models are designed to provide a natural staging scheme while also characterizing the uncertainty of the ordering of biomarkers without requiring a priori clinical diagnostic information or explicit biomarker threshold criteria.

Here, we report the application of a probabilistic event‐based model (EBM)^11^, ^12^ to ALS. Our aim was to adapt the EBM of ^12^ for application to ALS and here we describe that modeling process. We then tested the ALS EBM model on a specific imaging parameter as ‘proof of concept’; while previous EBM studies have modeled volumetric, cortical thickness, or connectivity changes, we chose fractional anisotropy (FA) derived from diffusion MRI, as this is the quantitative MRI modality that has, overall, revealed the most consistent changes in ALS to date.^16^ Although we selected the key white matter pathways for inclusion in our analysis based on existing neuroimaging studies, it is important to emphasize that, in keeping with this being an unbiased approach, we had no a priori hypothesis about the expected ordering of involvement of these cerebral white matter tracts.

## Materials and Methods

### Data description

Five datasets were available for use in this study. Set E was acquired on a GE Signa Horizon HDxt 1.5T clinical scanner (General Electric, Waukesha, WI) at the Brain Research Imaging Centre, University of Edinburgh between 2010 and 2012. The diffusion MRI protocol consisted of seven T_2_‐weighted (b∼0 s mm^−2^) and sets of diffusion‐weighted (*b* = 1000 s mm^−2^) whole brain single‐shot spin‐echo echo‐planar imaging (EPI) volumes acquired with diffusion encoding gradients applied in 64 noncollinear directions.^17^ The acquisition parameters were: (1) field of view 256 × 256 mm; (2) imaging matrix 128 × 128; and (3) 72 × 2 mm thick contiguous axial slice locations giving 2 mm isotropic voxels. The repetition and echo times for the single‐shot spin‐echo EPI sequence were 16.5 sec and 98.3 msec respectively.

Sets F and K were acquired between 1999 and 2011 at the Centre for Neuroimaging Sciences, King’s College London. Set F was obtained on a 1.5T GE Signa HDx system (General Electric, Waukesha, WI). The protocol included diffusion‐weighted EPI with diffusion gradients applied along 64 directions and maximum *b*‐value of 1300 s mm^−2^. Each volume was acquired using a multislice peripherally gated doubly refocused spin echo EPI sequence, from 60 contiguous near‐axial slice locations with anisotropic (1.875 × 1.875 × 2.5 mm) voxels. The echo time was 101.3 msec, while the effective repetition time varied between subjects in the range 12 and 20 RR intervals, depending on individual participants' heart rates. Set K was obtained on a 3T GE Signa HDx system (General Electric, Waukesha, WI) between 2008 and 2011. The protocol included diffusion‐weighted EPI, with diffusion gradients applied along 32 noncollinear directions and maximum *b*‐value of 1300 s mm^−2^. Each volume was acquired using a multislice peripherally gated doubly refocused spin echo EPI sequence, from 60 contiguous near‐axial slice locations with 2.4 mm isotropic voxels. The echo time was 104.5 msec, while the effective repetition time varied between subjects in the range 12 and 20 RR intervals, depending on individual participants' heart rates. Full image acquisition details are given in.^17^

Set N was obtained on a 1.5T Siemens Avanto system (Siemens AG Medical Solutions, Erlangen, Germany) at the Clinical Imaging Sciences Centre, Brighton and Sussex Medical School between 2014 and 2016. Multishell diffusion‐weighted images were acquired with single‐shot, twice‐refocused pulse‐gradient spin‐echo EPI, using three b‐values (nine directions with *b* = 300 s mm^−2^, 30 directions with *b* = 800 s mm^−2^, and 60 diffusion directions with *b* = 2400 s mm^−2^), optimized for neurite orientation dispersion and density imaging (NODDI).^18^ Ten nondiffusion weighted (*b* = 0) volumes were acquired. A parallel imaging (GRAPPA) speed up factor of 2 was used; echo time/repetition time = 99 msec/8400 msec; 2.5 mm isotropic voxel size. Full image acquisition details are given in.^19^

Set O was obtained on a 3T Siemens Trio scanner (Siemens AG Medical Solutions, Erlangen, Germany) at the Oxford Centre for Clinical Magnetic Resonance (OCMR) between 2009 and 2013, as part of the Oxford Study for Biomarkers in MND (“BioMOx”). The protocol included diffusion‐weighted whole‐brain EPI, with diffusion gradients applied along 60 isotropic directions and maximum *b*‐value of 1000 s mm^−2^; echo time/repetition time = 94 msec/10,000 msec; 2 mm isotropic voxel size. Four nondiffusion weighted (*b* = 0 s mm^−2^) volumes were acquired. Full image acquisition details are given in.^7^

Specific eligibility criteria for Sets E, F, K, N, and O are given in;^7^, ^19^, ^20^, ^21^, ^22^ at the time of enrollment, all patients were systematically screened for cognitive impairment. Only patients with a diagnosis of probable or definite ALS were selected for inclusion in this study. One patient was removed from Set E, due to fulfilling the criteria for possible behavioral variant FTD. Seven and three participants were removed from Sets F and K, respectively, due to poor image quality. Eighteen patients were removed from Set O due to having a diagnosis differing from probable or definite ALS. Two controls were removed from Set O due to their young age. Basic demographic and clinical characteristics of all five datasets as included in this study are summarized in Table 1.

**Table 1:** Demographics at time of neuroimaging for the five datasets E, F, K, N, and O.

	Demographics	Set E	Set F	Set K	Set N	Set O
Controls	*N*	30	22	24	23	29
	Gender (M/F)	16/14 (53%)	14/8 (64%)	19/5 (79%)	14/9 (61%)	14/15 (48%)
	Age at scan (years, mean ± SD)	59.1 ± 11.5	49.8 ± 15.6	47.3 ± 8.2	61.5 ± 9.3	52.5 ± 11.7
ALS	*N*	29	35	28	23	39
	Gender (M/F)	16/13 (55%)	20/15 (57%)	25/3 (89%)	16/7 (70%)	25/14 (64%)
	Age at scan (years, mean ± SD)	58.3 ± 11.3	54.0 ± 12.1	52.6 ± 11.8	64.3 ± 8.0	57.6 ± 10.5
	Age at onset (years, mean ± SD)	57.4 ± 9.9*	51.2 ± 13.4**	50.5 ± 11.8	62.0 ± 8.1	54.9 ± 11.0
	ALSFRS‐R score (mean ± SD)	38.8 ± 6.9	37.7 ± 6.6**	40.6 ± 4.1	40.0 ± 5.2	33.9 ± 5.4

* *N* = 28.

** *N* = 27.

### Magnetic resonance imaging analysis

All diffusion‐weighted EPI volumes were corrected for involuntary motion and eddy current distortions using affine registration and the FMRIB’s Linear Registration Tool (FLIRT), included in FSL 5.0.7, which is documented and available freely online (https://fsl.fmrib.ox.ac.uk). The images were skull‐stripped using FMRIB’s Brain Extraction Tool (BET). All datasets were manually inspected for low signal to noise ratio and movement artifacts.

For all diffusion imaging analysis, the single tensor (ST) model was used to derive FA measurements. The ST model was fitted with weighted least squares, using FMRIB’s *dtifit*.

Normalization into MNI space was performed using ANTs 2.1.0, which is documented and available freely online (http://stnava.github.io/ANTs/). A study‐specific template was created for each of the five datasets. Each subject’s FA map was warped to the corresponding template, and all templates were warped to the JHU ICBM‐DTI‐81 FA 1 mm atlas,^23^ included in FSL 5.0.7. These warps were combined to produce a single warp for each subject, which was then applied to their FA map.

To reduce the impact of scanner and site effects, the FA voxel data were harmonized using the ComBat statistical approach. ComBat is a batch adjustment method developed for genomics data and adapted for diffusion MRI.^24^ Age and patient/control status were included as covariates during the harmonization process.

A two‐tailed *t*‐test (equal variances) for the combined cohorts for the difference in mean age between controls and patients showed *P* = 0.053. A voxel‐based analysis was therefore performed to model and correct for the effects of age on FA, using the existing normalization. A generalized linear model was fitted to the FA data of controls only, using SPM12 (r7487). The effects of age were regressed out of the FA maps, and the mean regional values that entered the EBM were computed from the residual images.

### Event set

**Figure 1:**
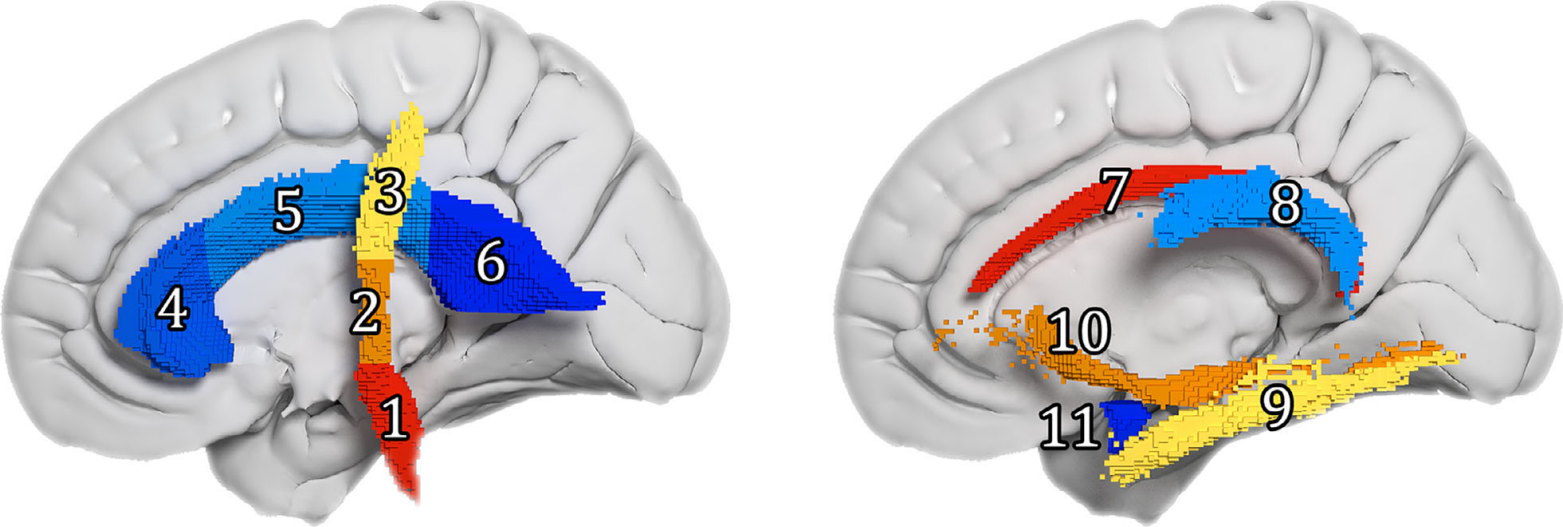
White matter regions selected as biomarker regions (only the left hemisphere portions are visible). 3D render of the ROI masks in MNI space; each cube is equal to a 1mm isotropic voxel. 1–3 = corticospinal tract inferior/middle/superior, 4–6 = corpus callosum genu/body/splenium, 7 = cingulum, 8 = superior longitudinal fasciculus, 9 = inferior longitudinal fasciculus, 10 = inferior fronto‐occipital fasciculus, 11 = uncinate fasciculus.

Seven white matter (WM) tracts were selected for analysis (Table 2, Fig. 1) based on their likely involvement in ALS pathology from analysis of the pathological and neuroimaging literature. ALS diffusion tensor imaging (DTI) studies have most consistently reported that finding decreased FA within the corticospinal tracts (CST).^16^, ^25^, ^26^ Multiple DTI studies have reported FA decreases in the corpus callosum (CC),^27^, ^28^, ^29^, ^30^, ^31^ with the strongest FA decreases appearing to be located in the middle‐posterior parts of the CC, which link the motor and premotor cortices.^30^

**Table 2:** White matter regions selected for analysis.

White matter region	Abbreviation	Illustration (Fig 1.)
Corticospinal tract	CST	
Inferior/middle/superior	Inf/Mid/Sup	1‐3
Corpus callosum	CC	
Genu/body/splenium		4‐6
Cingulum (dorsal section)	Cingulum	7
Superior longitudinal fasciculus	SLF	8
Inferior longitudinal fasciculus	ILF	9
Inferior fronto‐occipital fasciculus	IFOF	10
Uncinate fasciculus	UF	11

With the exception of the corpus callosum, all tracts were further subdivided by hemisphere. Biomarker readings were taken as the mean FA value of each region.

Other WM regions are less consistently reported in ALS DTI studies. Significant changes of DTI metrics within the CST, CC, and superior longitudinal fasciculus (SLF) have been found to correspond with a higher burden of upper motor neuron (UMN) involvement in sporadic ALS patients.^32^ Decreased FA has also been demonstrated in the uncinate fasciculus (UF).^33^

Diffusion tensor tractography techniques have demonstrated correlations between performance in cognitive tasks and DTI changes in the CC, CST, and major long‐range association tracts: the cingulum, inferior longitudinal fasciculus (ILF), inferior fronto‐occipital fasciculus (IFOF), and UF.^34^

For each fiber bundle, region of interest (ROI) masks were created from the JHU DTI‐based white‐matter tractography atlases^23^ included in FSL 5.0.7. The ROI masks for the CST, IFOF, ILF, and SLF were created from the JHU white‐matter tractography atlas, thresholded at 50%; all other ROI masks were created from the ICBM‐DTI‐81 atlas. FMRIB's *fslstats* was used to calculate mean FA values for each ROI, and these values were used as biomarker readings.

To allow the investigation of a directional progression of ALS WM neurodegeneration, the CST was split in the inferior to superior direction. The boundaries were set at boundaries of the posterior limb of the internal capsule as given in the ICBM‐DTI‐81 atlas (MNI *z*‐axis coordinates: CST inferior *z* ≤ −5, CST middle − 4 ≤ *z* ≤ 18 and CST superior 19 ≤ *z*). The CC was divided into three ROIs, consisting of body, genu, and splenium, using the JHU atlas boundaries.

Subdividing the CST and CC created a total of 11 WM regions, each of which comprised a tract ROI combined across both cortical hemispheres. To allow for investigation of bilateral asymmetry in ALS progression, all tracts apart from the CC were further subdivided by cortical hemisphere, giving a second set of 19 WM regions from which FA biomarker values were derived. The EBM was applied separately to both sets of biomarkers.

### The event‐based model

We estimated the most likely ordering of events and their uncertainty across the cohort, using a version of the EBM adapted for ALS. The EBM defines a disease as a series of events, where an event is the change of a biomarker from a “healthy” state to a “diseased” state. The cut‐off point determining this change for each biomarker is not determined a priori, but instead derived from the biomarker data during the modeling process. Full mathematical details of the EBM are given in^11^, ^12^ and briefly summarized below.

Fitting the EBM to the data requires evaluating the likelihood *P*(*S|X*) of a particular event ordering *S* given the data *X*. For biomarker *i* and patient *j*, this is achieved by fitting simple models for the likelihood function *P*(*x_ij_|E_i_*) on the measurement *x_ij_* given that event *E_i_* has occurred, and similarly *P*(*x_ij_|­E_i_*) on the measurement *x_ij_* given that event *E_i_* has not occurred. These simple models are derived from a two‐component Gaussian mixture model, fitted to each biomarker.

It is important to highlight some differences between our approach and previous applications of the EBM. First, we applied the model to data from ALS patients. The ALS patient population is clinically distinct from the control population; this may not apply to dementia, in which there is likely to be a continuum in biomarker distribution between normal aging and symptomatic disease.^35^ This distinction allowed for constraining the mixture fitting: a Gaussian distribution was fitted to the control data, and the 95% confidence intervals of the resulting parameters were used as constraints for the first mixture model component. The second model component and mixing proportion were left unconstrained. Following the fitting, the components were separated and used to model *P*(*x_ij_|­E_i_*) and *P*(*x_ij_|E_i_*) respectively.

Second, we used FA as a biomarker, as opposed to volumetric and cortical thickness measures^11^, ^12^, ^15^ or connectivity.^36^ The EBM is potentially sensitive to individual variation of biomarker readings, due to its dependence on Gaussian mixture models, the fitting of which can be biased by the presence of outliers. This is particularly important when using biomarkers such as FA, which is susceptible to noise and varies between anatomical regions.^37^ To reduce the effects caused by outliers, the mixture models were fitted 1000 times from bootstrapped samples. Samples for which the mixing proportion had collapsed to 0 or 1 were then excluded to avoid biologically unrealistic solutions. The mixing proportion of ILF (right hemisphere) was constrained to be >0.5, in order to prevent consistent fitting failure. To obtain the final mixture model parameters, the median of the remaining bootstrapped parameters was taken. This is an addition to the EBM which was not used in previous applications.

Following the fitting of the mixture models, estimation of the most likely ordering of events (the “inferred event order”) and characterization of their uncertainty was performed using a Markov chain Monte Carlo (MCMC) algorithm to sample from the posterior event distribution *P*(*S|X*). We note that the MCMC sampling was performed on only the patient biomarker data, as justified by the clinically distinct control and patient populations of ALS.

Finally, cross‐validation was performed as described in ^12^, ^15^ with the mixture models and the most likely event sequence re‐estimated for a further 10,000 bootstrap samples. Two further weak constraints were used during cross‐validation: mixing proportions were constrained to be ≥ 0.01 and ≤ 0.99, and for the second model component, the standard deviation σ was set to be ≥ 0.001. For each sample, the mixture models were directly fitted without further bootstrapping, in order to minimize the risk of underestimating the cohort variability. Cross‐validation overstates the uncertainty of the inferred event order, giving a more conservative picture than that of the MCMC samples.

### Ethics approval

All participants gave written informed consent at inclusion, in accordance with the Declaration of Helsinki. Participants in Sets E, N, and O explicitly gave consent for their data to be used in further studies. We obtained additional ethical approval to use participant data from Sets F and K in this study from NRES Committee London – Stanmore (REC reference 14/LO/1484).

## Results

### Inferred order of events – combined hemispheres

**Figure 2:**
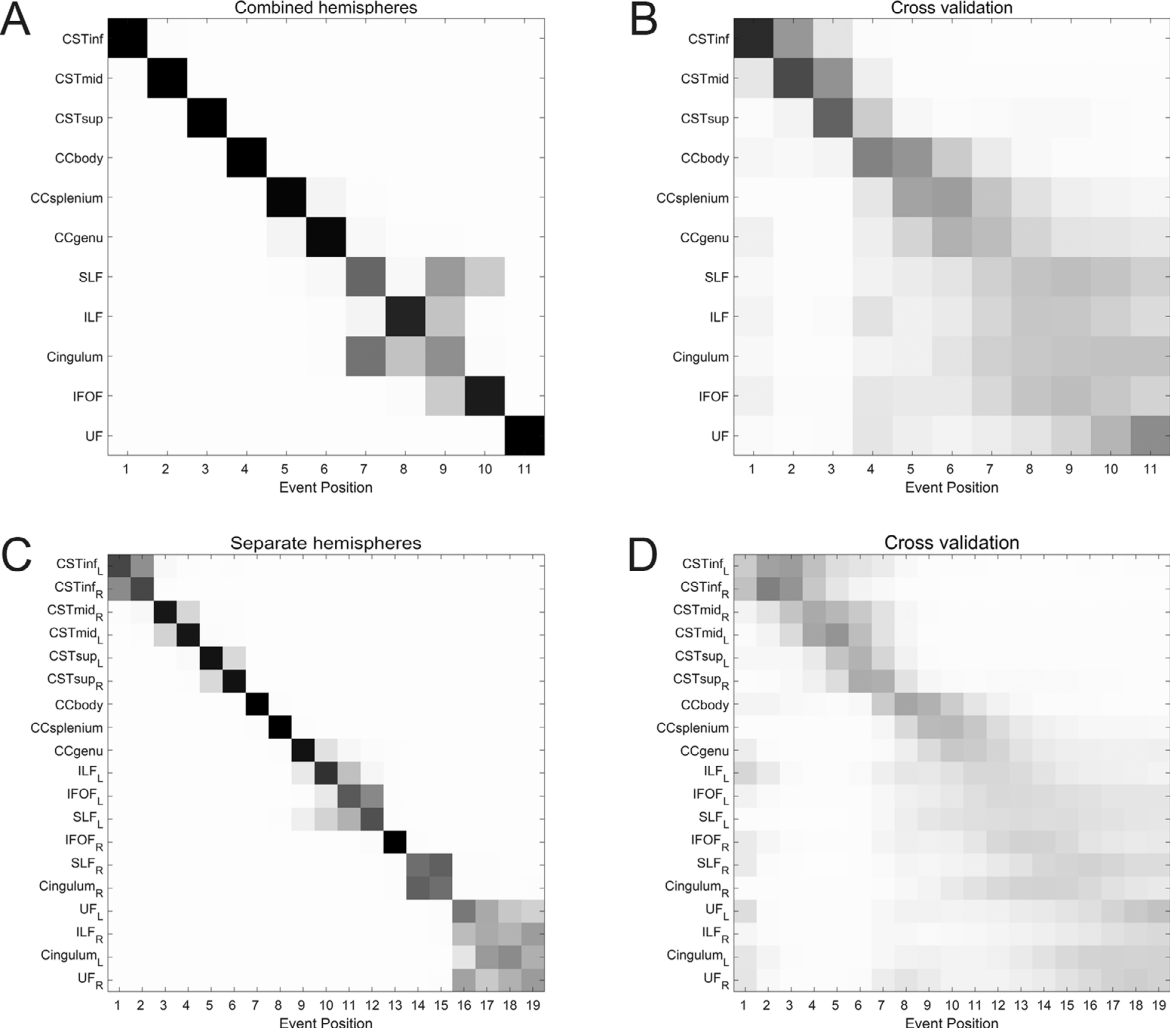
Event‐based models of amyotrophic lateral sclerosis progression. Positional variance diagrams (PVDs) for (A,B) 11 white matter regions (combined across cortical hemispheres) and (C,D) 19 white matter regions (subdivided by cortical hemisphere). The inferred event order is given along the y‐axis (top to bottom). The grayscale intensity of each square is proportional to the posterior confidence with which a biomarker occupies a position in the event sequence; higher intensity corresponds to lower positional variance and thus greater confidence. Left: PVDs of MCMC samples from the event‐based model. Right: PVDs from cross‐validation through bootstrapping. These diagrams overstate the uncertainty of the inferred event order, giving a more conservative picture than that of the MCMC samples. CST = corticospinal tract, CC = corpus callosum, Cingulum = cingulum (dorsal section), IFOF = inferior fronto‐occipital fasciculus, ILF = inferior longitudinal fasciculus, SLF = superior longitudinal fasciculus, UF = uncinate fasciculus. Inf/mid/sup = inferior/middle/superior, L/R = left/right hemisphere.

Figure 2A is a positional variance diagram, showing the inferred order of events on the *y*‐axis (top to bottom), and each event's variation across the MCMC samples. This variation may be considered to represent the uncertainty of an event's ordering, and is represented by the intensity of each square: higher certainty corresponds to darker squares.

The event sequence shows with high confidence that the earliest changes detected are in the CSTs and CC body; the distal CST is more susceptible to MRI degenerative change than the proximal segment as reflected by changes in FA in the three segments of the CSTs. The next regions affected are the CC splenium and body, which are followed by a cluster comprised of the SLF, ILF, and cingulum. The final ROIs to be affected are the IFOF and UF.

We note that the inferred event sequence shows fibers ordered by type; projection fibers are the first affected, followed by commissural fibers, and finally long association fibers.

Cross‐validation reveals a similar picture, with increased uncertainty across all biomarkers (Fig. 2B). The CST biomarkers are the events with the highest positional confidence, reflecting the CST’s role as the tract most consistently implicated in ALS DTI studies.

### Inferred order of events – separate hemispheres

Qualitatively, the inferred order when considering hemispheres separately (Fig. 2C) is broadly similar to that for the combined hemispheres (Fig. 2A), although there are differences within the ordering of the association fibers. Again, the distal over proximal degeneration of the CSTs is apparent, with the event sequence not favoring one hemisphere over another; i.e., showing no clear evidence for bilaterally asymmetric progression within the CSTs. We note that ILF_R_ required an extra constraint during mixture fitting to avoid consistent fitting failure; the need for this constraint may indicate reduced accuracy of temporal staging for this biomarker.

As before, cross‐validation (Fig. 2D) shows similar event positional variance, albeit with increased uncertainty across all biomarkers; the positional uncertainty is greatest for the long association fiber regions and the extremities of the CC, qualitatively corresponding with the increased variability seen in Figure 2B.

### Imaging biomarker staging

**Figure 3:**
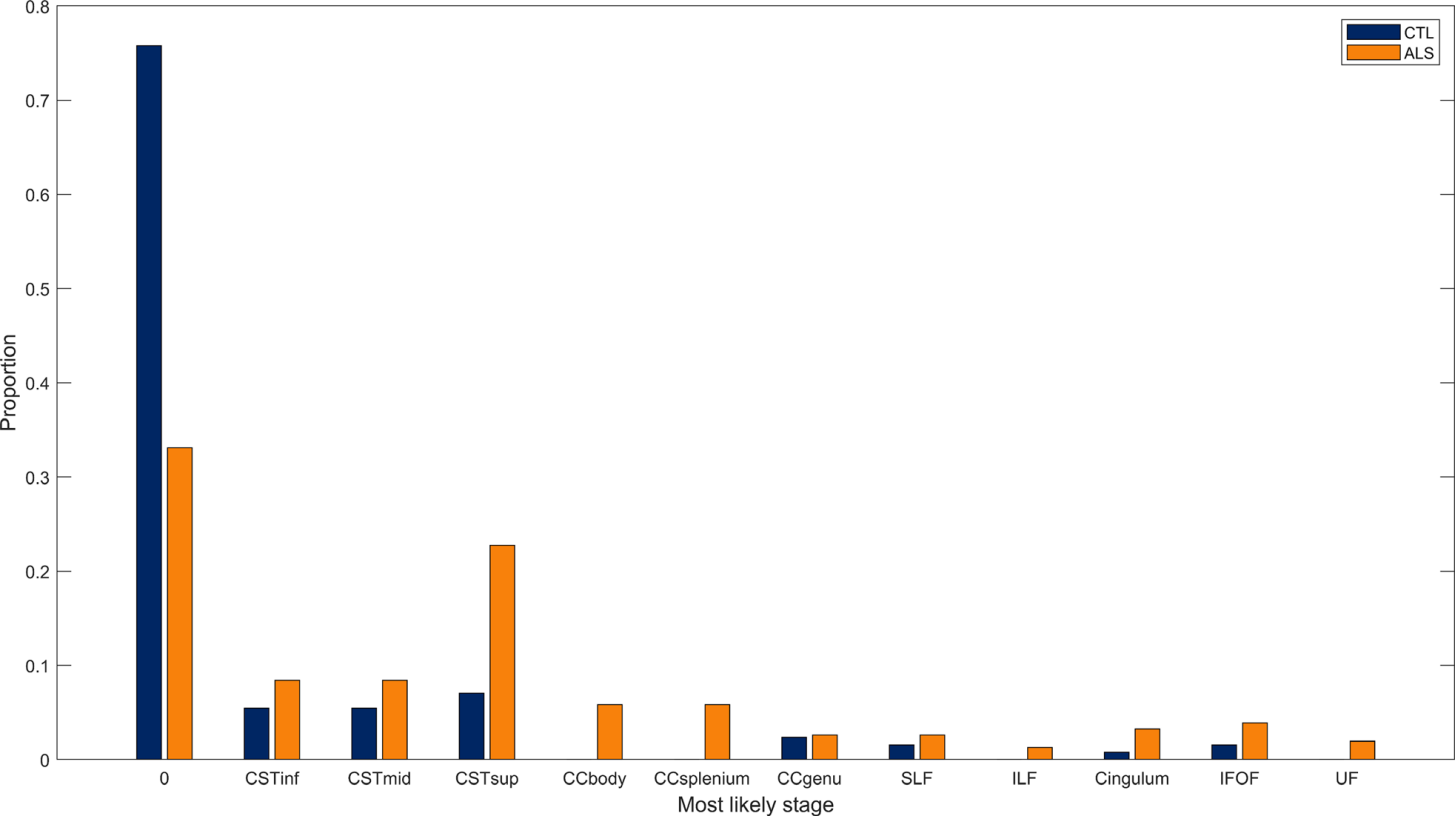
Proportion of patients and controls allocated to each biomarker stage by maximum likelihood. The stages are in the same order as given in Figure 2A.

The EBM also possesses fine‐grained staging capabilities,^12^, ^15^ whereby each person is assigned a biomarker stage that best reflects their measurements. In order to maximize the accuracy of stage assignment, we applied the staging process to the combined hemisphere biomarker set, as this has the most distinct event ordering*.* The staging proportions for all participants are shown in Figure 3, differentiated by control/patient status. Unlike previous applications of the EBM to Alzheimer’s disease, we do not find strong separation between the diagnostic groups (i.e., patients and controls) when performing this subject‐specific staging. We note that most previous applications of the EBM leave out biomarkers that do not show statistically significant differences between patients and controls. However, our aim here is more to elucidate the sequence of change than to stage patients so we retain all biomarkers, but acknowledge that the ordering may not be reliable for markers that do not discriminate patients and controls.

## Discussion

Using an event‐based probabilistic model developed for ALS on the basis of similar approaches in Alzheimer’s disease and Huntington’s disease,^11^, ^12^ we infer a temporal susceptibility of MRI‐based pathological involvement in key white matter pathways in ALS, using FA as a biomarker of axonal damage.

Our main findings can be summarized as follows. First, the model suggests that the earliest detectable MRI‐based intracerebral white matter FA changes are in the CSTs. Second, FA changes in the CSTs may have a directional susceptibility, with FA changes occurring distally prior to proximally. Third, the CC is involved next in sequence to the CSTs, with detectable FA changes occurring in the CC body, then in the splenium and finally in the genu. Fourth, the long association fiber tracts are affected after the CSTs and CC body. Fifth, we have found that subject‐specific staging reveals a significant overlap between controls and ALS patients.

Regarding the CSTs, all longitudinal MRI studies to date identify this as the earliest white matter tract in which MRI‐based abnormalities are detectable,^16^ in keeping with the outcome of our EBM. However, our observations suggest that the pathological dynamics affecting the CSTs is a ‘dying‐back’ process or distal axonopathy.^38^ Classical histopathology cannot, by definition, identify dynamic aspects of CST degeneration, but in accord with our findings pathological changes along the proximal‐distal are usually more evident in distal compared to more proximal portions of the pathway,^39^ and in keeping with a so‐called ‘dying‐back’ process. There has been much debate as to whether the degenerative process is driven by changes in the primary motor cortex as an anterograde form of neurodegeneration or is retrogradely initiated from somatic motor neurons.^40^ However, this dualism is likely to be misleading, since the perikaryon, dendrites, and axon form a unified functional and, presumably, pathophysiological unit. Data‐driven approaches to old controversies may help to resolve this issue, if indeed it is a meaningful dichotomy.

Likewise, involvement of the CC has been a consistent finding in MRI studies in ALS.^7^, ^30^, ^32^ In the EBM, the staging of CC involvement evolved from the body, to the splenium and the genu. This is in keeping with the notion that the callosal fibers most affected in ALS are those that connect the primary motor areas of the hemispheres.^41^ The EBM results are broadly keeping with the Braak staging system^5^ regarding the CSTs and CC. However, in contrast with recent studies^6^, ^8^, ^26^ that have sought to confirm this pathological staging system using cross‐sectional and longitudinal neuroimaging data, our EBM makes no a priori assumptions on the ordering of white matter involvement.

Turning to the other white matter tracts included in our modeling: the cingulum, SLF, ILF, IFOF, and UF have all been identified as showing changes in DTI metrics in ALS.^32^, ^33^, ^34^ Furthermore, their involvement has been linked to cognitive changes across several domains including attention and executive functions,^22^, ^34^, ^42^ and language processing (the ILF, IFOF and UF).^42^, ^43^, ^44^, ^45^ The ordering of these long association fibers comprises the main differences between the inferred event orders for combined and separate hemispheres (Fig. 2A and C). The involvement of these tracts in ALS DTI studies is less consistently reported than the CSTs and CC,^16^, ^25^, ^26^, ^27^, ^28^, ^29^, ^30^, ^31^ suggesting a smaller effect size and greater heterogeneity of FA values within the long association fiber ROIs; the differences in inferred event orders may be reflective of this. An alternative explanation is the reduced signal‐to‐noise ratio in the case of separate hemisphere ROIs.

Our selection of white matter tracts could be criticized in that we did not include in our EBM specific pathways that have been prioritized on the basis of the pathologically based hypothesis of disease spread.^5^, ^6^, ^26^, ^46^ We had reservations about the self‐fulfilling nature of the selection based on this hypothesis and chose to focus initially on the pathways identified in studies not based on this presupposition.^7^, ^32^, ^34^ However, it will be important to add these pathways to the model in future applications and developments of the EBM.

The mean FA values were extracted using atlas‐defined ROIs; this approach relies on registration to standard space and does not account for individual anatomical differences. An alternative approach is to use tractography to reconstruct the tracts of interest on a participant‐wise basis. Tractography, however, is strongly affected by data acquisition parameters, such as maximum b‐value and number of diffusion directions. Given the variability in the acquisition protocols used for the datasets included in this study, we opted for minimizing the bias using predefined ROIs.

As well as finding the most likely event order, the EBM is capable of staging disease progression in fine detail. Unlike previous applications of the EBM to Alzheimer’s disease, we find poor separation between the diagnostic groups (i.e., patients and controls) when performing this subject‐specific staging. The assignment of controls to late biomarker stages is likely due to the limited sensitivity and specificity of FA as a biomarker for ALS.^16^ DTI is limited in its sensitivity to tissue microstructure^47^ and more sophisticated approaches, such as NODDI, could improve on these results. However, as this study relied on historical data it was not possible to use such approaches. Future studies employing biomarkers with greater diagnostic accuracy should be able to demonstrate improved separation between ALS patients and controls.

By setting Stage 1 as the cut‐off point (i.e., Stage 0 = healthy, Stage 1 and above = ALS), the subject‐specific staging distinguished between patients and controls with a sensitivity of 66.9% and specificity of 75.8%. This is comparable to that of other FA‐based ALS biomarkers.^48^ The proportions of participants assigned to various biomarker stages are also informative: Figure 3 shows that 33.1% of patients are assigned to biomarker stage 0, equivalent to none of the included biomarkers being in a diseased state. While the heterogeneity of ALS should be considered as a causal factor, another plausible explanation is that relevant biomarkers have not been included in this analysis; i.e. this biomarker staging could suggest that detectable changes in the mean FA of the CSTs do not represent the beginning of ALS pathology.

The EBM method is subject to the difficulties inherent in any data‐driven approach: the model finds patterns within the data, and these patterns require interpretation within the context of the pathology of the disease. Moreover, the probabilities are based on the variable sensitivity of water diffusion‐based metrics, which are known to vary physiologically along the length of many tracts, and are influenced by crossing fibers.^49^ Any interpretation must be subjective to a certain degree, particularly when establishing the biological significance of the modeling output, given that the precise relationship between MRI metrics and histology is still being uncovered.^50^ Likewise, as with any modeling process, simplifications and assumptions about the disease process are unavoidable. In particular, the EBM assumes that the event sequence is consistent over all patients, which is unlikely to hold for a heterogeneous disease such as ALS. We have tried to mitigate the impact of disease heterogeneity by using bootstrapping to fit the mixture models, thus reducing the effects of individuals and giving an “average disease progression” across the entire cohort.

As with any data‐driven method, sample size and heterogeneity must be taken into consideration when drawing conclusions; the clinical cohorts available to us were relatively small, which warrants caution when generalizing results beyond this study. While the cohorts show differences in demographic and prognostic characteristics, especially age and gender distribution, such variations are unlikely to invalidate the analysis of tract involvement in the context of EBM but might contribute to greater variability in the ordering of events. It is therefore all the more striking that the ordering of change in FA along the CSTs is highly consistent, as is the involvement of the CC. Finally, historical cohorts such as ours pose challenges derived from older technology and lack of standardization of scanning protocols between cohorts.

In summary, we have adapted the EBM and applied it to five independent ALS cohorts, using FA as the most robust measure of white matter damage in this disease. The model clearly and consistently demonstrates the dynamic of pathological involvement in ALS, which is in keeping with known pathological processes derived from more subjective approaches.^7^ Having shown here that the EBM can be applied to a relevant biomarker in ALS, the next stage will be to take a more inclusive approach incorporating a much wider range of biomarkers. The field of data‐driven modeling applied to progressive neurological diseases is relatively new, but this study indicates that the EBM has great potential to inform understanding of the dynamics of the underlying biological processes in ALS.

## Author Contributions

Study design and conception: MCG, PNL, MC, DCA, and ALY. Acquisition of data: RB, SA, MB, RALM, AAC, LHG, ST, and MRT. Drafting the manuscript and preparing figures: MCG, PNL, and MC.

## Conflict of Interest

Gabel and Broad report grants from Motor Neurone Disease Association, during the conduct of the study.
